# Editorial Correspondence

**Published:** 1879-03

**Authors:** A. J. Shaffer

**Affiliations:** Gainesville, Ga.


### EDITORIAL CORRESPONDENCE.

GAINESVILLE, GA., Feb. 20, 1879.

*Dear Dr. J. G. Westmoreland:* As the nosology pathology and treatment of that terrible malady, diphtheria, now becoming common with children, is exciting much attention throughout the medical world, I contribute my mite. Each writer in regular order differs from others in treatment, and this is proof that the true nosology and pathology of the disease is yet a mooted question; and hence the treatment is different by every physician who may be called upon to treat the disease. In '58–9 and again in '67, at which dates it was quite prevalent in Gwinnett county, I followed the treatment suggested by the best practical medical men of the country, taking it for granted that it was a local disease of the tonsils. This local treatment was very unsatisfactory. By close observation I was forced to the conclusion that the disease was constitutional, manifesting itself by local lesions. I

changed my treatment in accordance with this view of the disease, which change I have not yet repented of. In 1877 it prevailed to an alarming extent in and along the lines of Hall and Gwinnett counties. I prescribed for a great many cases, and not one case that used the following prescription ended fatally :

To a child six years old I gave six grains of sulphate of quinia and four drops of hydrochloric acid every hour until the symptoms gave way, diminishing or increasing according to the age of the patient. In a large majority of cases the grave condition was improved, and the little patient passed into a quiet, refreshing sleep.

℞—Sulph. of quinia gr. 48
Hydrochloric acid m. 32
Water f℥j.

Mix.

Sig.—One teaspoonful to be given every hour until the grave symptoms give way.

The following is the easiest way to give the medicine and more palatable for a child :

℞—Sulph. of quinia gr. vi.
Acid hydrochloric m. iv.
Honey, half teaspoonful.

Mix.

Sig.—Repeat the dose every hour in severe cases until the symptoms give way.

Yours truly, A. J. Shaffer, M.D.

